# Micro- and Nanocrystalline NiO Synthesized by Joule Heating and Thermal Oxidation Methods: A Comparative Study

**DOI:** 10.1021/acs.cgd.4c01439

**Published:** 2025-02-06

**Authors:** Diego J. Ramos-Ramos, G. Cristian Vásquez, David Maestre

**Affiliations:** Departamento de Física de Materiales, Facultad de CC. Físicas, 16734 Universidad Complutense de Madrid, Madrid 28040, Spain

## Abstract

The high stability and intrinsic p-type nature of nickel oxide (NiO) make it an interesting material for modern oxide-based technology microdevices. Nowadays, the industry demands more sustainable, highly efficient, and low-energy-consumption synthesis routes as an alternative to conventional methods that commonly involve high temperatures for long times or complex chemical routes. In this work, a fast, low-cost, and energy-saving synthesis based on the Joule heating (JH) process has been employed for the fabrication of micro- and nanocrystalline NiO. The as-grown NiO samples have been investigated as a function of the growth parameters, and special attention has been paid to the differences and similarities between microcrystals grown by JH or vapor–solid (VS) thermal treatments. In particular, Raman spectroscopy reveals that the JH process results in a very reproducible and controllable NiO microcrystalline structure as compared to VS regardless of the fast oxidation process. Cross-sectional analysis of the NiO grown by JH confirms the presence of inner/outer regions with variable microstructure, composition, and physical properties as a function of the different oxidation conditions promoted during the JH process. The mechanisms underlying the JH process have been discussed and compared with those related to the VS method.

## Graphical abstract

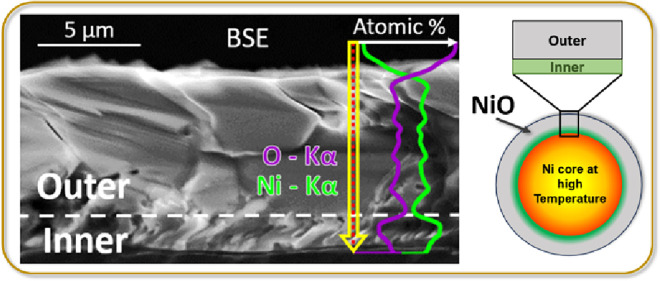

## 1 Introduction

Nickel oxide (NiO) stands out among the very few p-type wide band gap oxides owing to its interesting range of applicability in photovoltaics, sensing, energy storage, and electrochromic devices,^1–9^ to name a few. Despite the appealing properties of this versatile oxide, its use still lags in performance in various fields of technological research, as challenges still need to be overcome in the control of the employed synthesis process and the understanding of some of the characteristic properties of NiO. Nickel oxidation has been investigated for decades as a reference system for metal oxidation and corrosion studies; however, insights into the oxidation mechanisms are yet to be widened as this system is continuously revisited. Besides, thermal oxidation of Ni in a furnace under ambient or oxidizing atmospheres, as well as thermal evaporation processes based on Ni precursors, has been employed so far for the growth of NiO at the micro- and nanoscale. As an example, NiO microcrystals and wires grown by a vapor–solid (VS) method at 1000–1400 °C for more than 10 h, under a controlled atmosphere, have demonstrated potential applicability as gas sensors and optical microresonators,^10,11^ among others, owing to their physical properties, variable Ni^3+^/Ni^2+^ ratio, specific dimensions, and morphology. However, most of these thermal methods based on Ni oxidation require extended treatments and high temperatures, thus involving high energy consumption, while other common physical synthesis routes, such as sputtering, pulsed laser deposition, chemical vapor deposition, or molecular beam epitaxy, among others, usually demand high vacuum technologies as well.^12^ In most of those cases, not only high energy consumption but also high technology requirements and considerable economical expenses are involved. Among the chemical routes employed for the synthesis of NiO at the micro- and nanoscale, wet-chemistry approaches commonly demand various subsequent steps and controlled reactions, which make them laborious and time-consuming.^13^ Besides, in some cases, toxic precursors or nonintentional secondary products are involved; therefore, some alternatives, such as “green synthesis” routes, are considered.^1^ In response to the worldwide energy demand and ecological concerns, alternative clean and affordable synthesis methodologies based on low-energy consumption, sustainability, and preventing the use of toxic and critical elements are increasingly required.^12,14,15^

In this work, a rapid, economic, and high-efficiency method based on resistive heating has been used for the synthesis of micro- and nanocrystalline NiO as an alternative to the conventional chemical routes and thermal treatments. This method, named Joule heating (JH), is based on the heating of a metallic nickel wire using a high current flow via the Joule effect^16,17^ under ambient conditions. This JH method promotes a notably fast oxidation of the metallic wire, thus avoiding the use of high-temperature furnaces, catalysts, and vacuum chambers, which are usually required in other synthesis methods.^12^ Actually, not only are large energy savings promoted, but also the economical consumption can be drastically reduced by using JH methods as compared to VS ones. Importantly, the JH method allows for the growth of NiO microcrystals with similar crystalline morphology and, potentially, the same applicability as samples synthesized by a thermal vapor–solid method (VS), but it uses a rather fast and energy-saving growth method with strong scaling-up potential. The JH method provides the necessary flexibility to precisely adjust the growth parameters during the synthesis process. However, a deeper understanding and control of the structural defects that result from the JH process and the underlying physical mechanisms, which involve electromigration and ionic diffusion, are still required to tailor and widen its applicability in the fabrication of oxide-based low dimensional structures. To proceed, in this work, diverse microscopy and spectroscopy techniques have been used for the in-depth analysis of the NiO micro- and nanostructures fabricated by JH. The impact of different growth conditions, such as time and applied current, on the morphology and properties of the NiO samples fabricated via JH have been also studied, in analogy with similar samples synthesized by a VS process in a furnace at high temperatures.

## 2 Experimental Section

### 2.1 Synthesis Methods

A 0.25 mm diameter metallic nickel wire (GoodFellow, 99.0% purity) was used as a precursor to fabricate NiO microstructures by the JH method under atmospheric conditions. Figure 1a shows an image and the corresponding scheme of the setup employed for the JH treatments using 6–7 cm long Ni wires previously cleaned in ethanol. The applied current was controlled with a Keithley 2400 Series SourceMeter, while the temperature reached was measured with an Infratherm pyrometer (IMPAC IGA 12/S Luma Sense Technologies GmbH). The temperature was calibrated at the center of the wire and near the electrodes, as shown in Figure 1b, where data measured near the electrodes are marked with an asterisk. The temperature was estimated as a function of the continuous applied current by increasing the current in different steps from 3.5 up to 6.3 A during 13 min and keeping it constant in each step for 1 min to stabilize the pyrometer measurement. The corresponding fitting curve is included as an inset in Figure 1b. It is important to mention that the wire melts at a current of 6.3 A, limiting the maximum range of the applied current during the JH experiments. At that point, according to the pyrometer measurement, the melting temperature is around 1400–1500 °C, in very good agreement with the Ni melting point of ∼1450 °C.^18^

**Figure 1:**
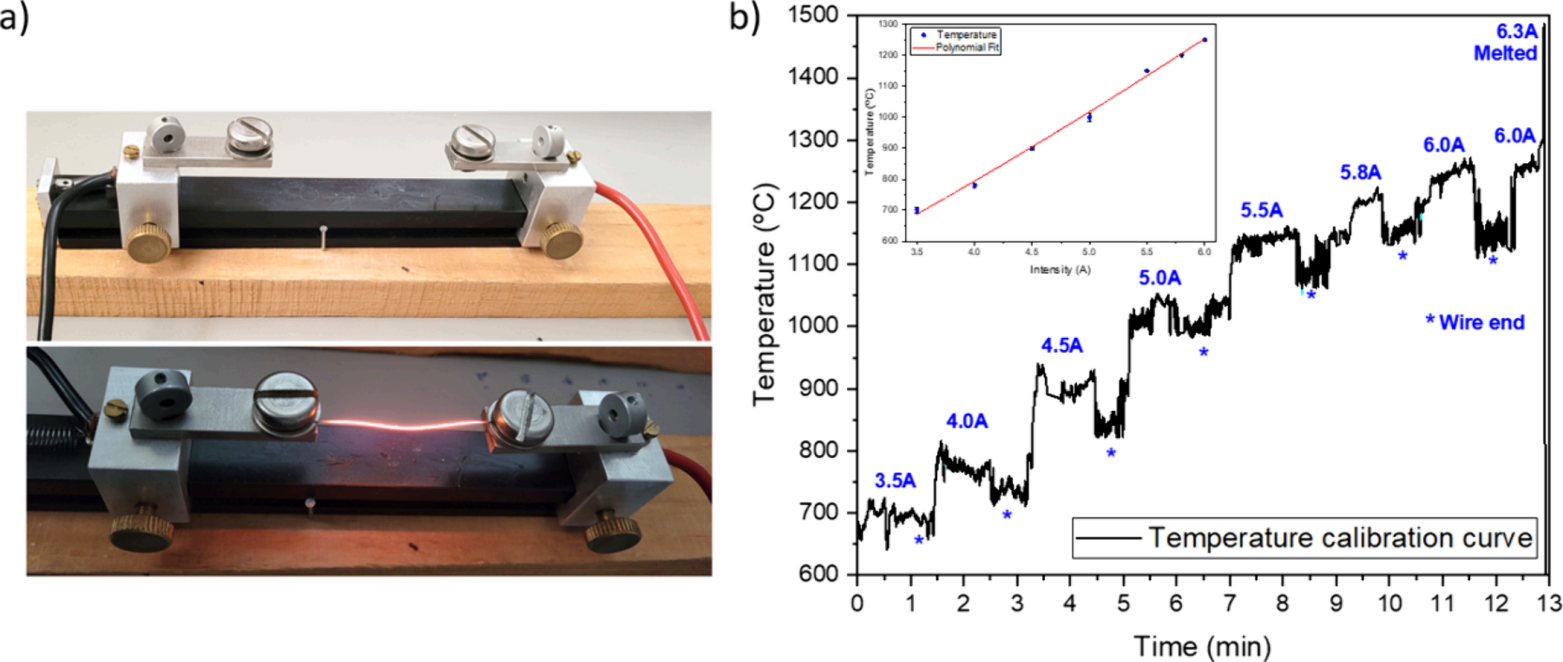
(a) Image of the Joule heating setup. (b) Calibration curve for the current–temperature estimation.

The list of samples synthesized by JH and the parameters used during the synthesis process is summarized in Table 1. The samples have been studied in-depth as a function of the applied current, which corresponds to an estimated temperature based on the calibration curve of Figure 1b and the duration of the treatment. The applied current ranges from 3.5 to 6 A, which involves current densities from 7.1 to 12.2 × 10^3^ A/cm^2^, while the duration of the JH process extends from 5 s to 10 min. To avoid variations associated with a lower temperature near the electrodes, the measurements included in this work correspond to regions around the middle area of the treated wires.

**Table 1:** List of Samples Synthesized by Joule Heating (JH) and Their Corresponding Growth Parameters (Applied Current and Duration Time)^a^

**sample**	**current (A)–temperature (°C)**	**duration**
JH-600-3m	3.0 A–600 °C	3 min
JH-900-3m	4.5 A–900 °C	3 min
JH-1150-5s	5.5 A–1150 °C	5 s
JH-1150-30s	5.5 A–1150 °C	30 s
JH-1150-3m	5.5 A–1150 °C	3 min
JH-1150-5m	5.5 A–1150 °C	5 min
JH-1150-10m	5.5 A–1150 °C	10 min
JH-1200-3m	5.8 A–1200 °C	3 min
JH-1250-3m	6.0 A–1250 °C	3 min

a The estimated temperature during growth has been included.

To analyze the main differences and similarities between the samples fabricated by the JH method and those synthesized by the vapor–solid (VS) process, additional reference samples were fabricated by sintering compacted metallic Ni powder (Sigma-Aldrich 99.99% purity, size <150 μm) in a muffle furnace (Carbolite RHF 1500) at temperatures from 1100 to 1300 °C for 10 h, similar to treatments reported in a previous work by Taeño et al.^5,19^ This conventional thermal treatment involves high temperatures and extended durations, thus involving a large energy consumption together with the presence of a controlled atmosphere during the growth process. To ease the sample comparison, the VS synthesis has been performed at temperatures close to those reached by the JH based on the calibration curve of Figure 1b. The reference samples grown by the VS method are indicated in Table 2.

**Table 2:** List of Samples Synthesized by the Vapor–Solid Method (VS), Similarly to Those Reported in a Previous Work by Taeño Et Al.^19^

**sample**	**temperature (°C)**	**duration**
VS-1100	1100 °C	10 h
VS-1200	1200 °C	10 h
VS-1300	1300 °C	10 h

### 2.2 Characterization Techniques

The morphological characterization of the NiO micro- and nanostructures was performed by scanning electron microscopy (SEM) in a Thermofisher Prisma-E SEM operated at 20 kV. The crystalline structure of the samples was assessed by X-ray diffraction (XRD) using a Bruker D8 Advance instrument with Cu Kα radiation (λ = 1.54158 Å) in Bragg–Brentano configuration. Raman spectroscopy was performed in a Horiba Jobin-Yvon LabRaman Hr800 confocal microscope (Horiba, Kyoto, Japan) equipped with a motorized stage for hyperspectral μ-Raman analysis using a 633 nm focused He–Ne laser (ca. ∼1 μm spot size) as the excitation source. Compositional mappings and energy-dispersive X-ray spectroscopy (EDS) spectra were acquired by using a Thermofisher Prisma-E SEM equipped with an UltraDry EDS detector operated at 20 kV.

## 3 Results and Discussion

During the synthesis process via JH, a noticeable change in the color of the Ni wire surface is observed. Initially, the Ni wire exhibits a characteristic gray-metallic color. However, upon completion of the thermal treatment by applying a current, the color of the wire changed to an intense greenish to black tone. This transition from a metallic color to green/black during the thermal treatment is consistent with the expected oxidation of nickel during the JH process under the presence of atmospheric oxygen.^20^

### 3.1 SEM, XRD, and EDS Analysis

Initially, reference NiO samples fabricated by a VS process using metallic Ni powder as the precursor were analyzed. Figure 2a,b shows SEM images from samples grown at 1100 and 1200 °C, respectively, for 10 h under a controlled Ar flow as representative samples fabricated by the VS process. It results in NiO microcrystals with sizes in the range of 5–10 μm that can exhibit pinholes and a terraced appearance. The morphology, structure, and optical properties of similar NiO microcrystals grown by a VS method, which strongly depend on the parameters employed during the thermal annealing, were reported in a previous work.^19^

**Figure 2:**
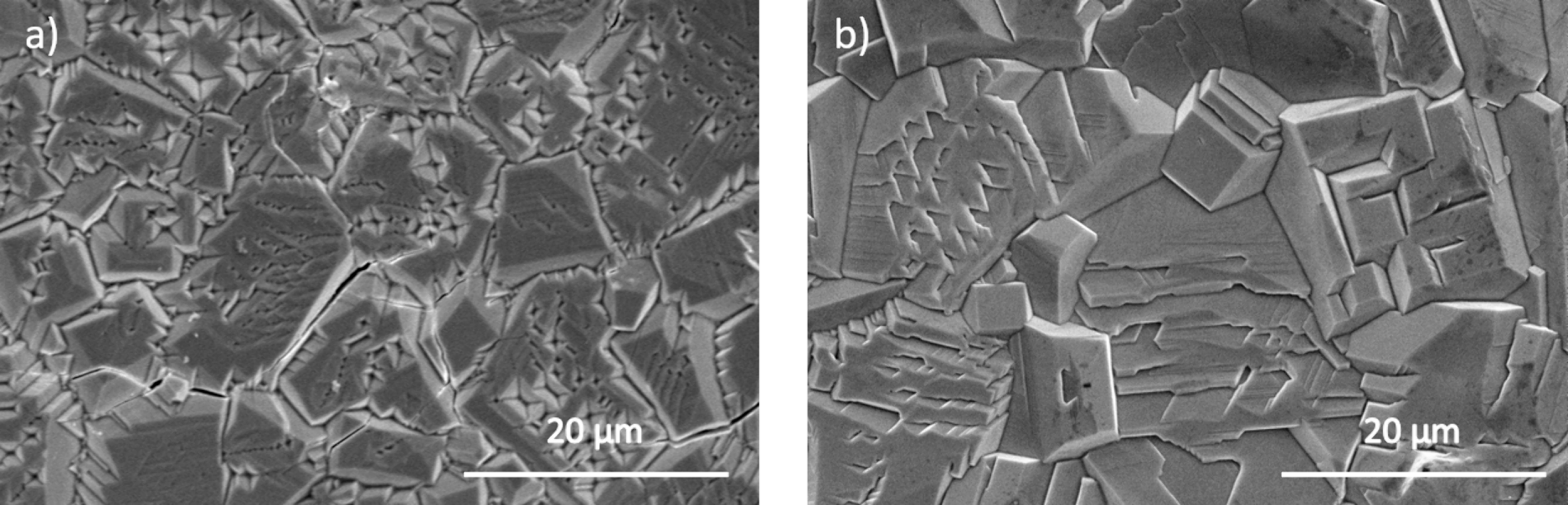
SEM images from reference samples grown by a VS method: (a) VS-1100 and (b) VS-1200.

The JH samples were analyzed as a function of the maximum current applied and the treatment duration. For reference, a SEM image of the metallic Ni wire prior to the JH treatment is shown in the Supporting Information (Figure S1).

First, samples fabricated by JH using currents from 3 to 6.0 A for 3 min were analyzed by SEM (Figure 3). According to the calibration curve (Figure 1b), for this range of current values, the Ni wire reaches temperatures ranging from 600 to 1250 °C.

**Figure 3:**
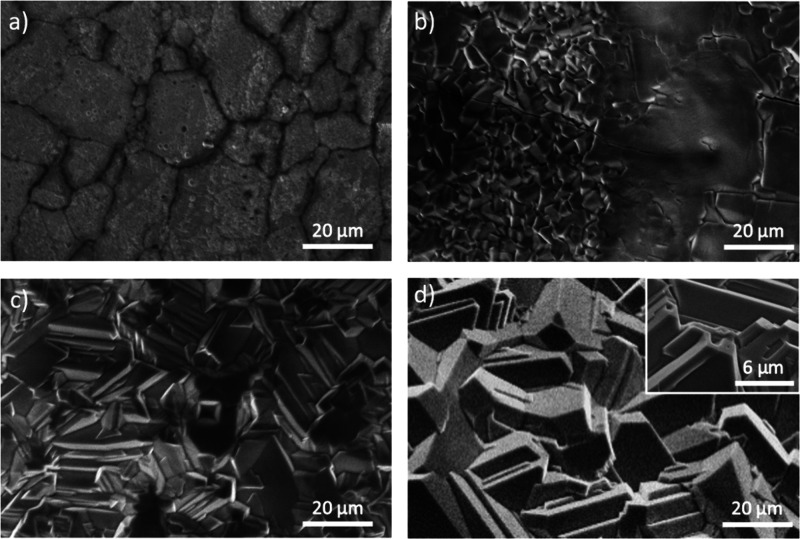
SEM images from samples (a) JH-600-3m, (b) JH-900-3m, (c) JH-1150-3m, and (d) JH-1250-3m synthesized at variable currents and a fixed duration of 3 min.

At a current of 3 A, corresponding to ∼600 °C, we can observe the initial oxidation stages leading to the formation of large domains of tens of micrometers width with well-defined boundaries (Figure 3a), similar to those observed in the untreated wire (Figure S1) but with a rougher appearance. According to some authors,^21,22^ the full oxidation of NiO is achieved in this range of temperatures. By increasing the applied current up to 4.5 A, corresponding to ∼900 °C, two types of morphologies can be observed (Figure 3b). Some regions show a flat appearance and large grains with dimensions of tens of micrometers, while other regions start to show slightly faceted grains with dimensions in the submicrometer range, as observed in Figure 3b, which indicate the initial stages of microcrystal formation. As the current is increased up to 5.5 A, which corresponds to ∼1150 °C, the surface of the treated wire becomes fully covered by microcrystals with dimensions of few microns, most of them exhibiting well-faceted surfaces and a terraced appearance (Figure 3c) with similar features as the observed by VS treatments (Figure 2). The formation of microcrystals with average sizes of 5–10 μm is observed by using current values of 6 A corresponding to ∼1250 °C (Figure 3d). In this case, microcrystals have similar features as those observed for the samples grown at 5.5 A, with well-faceted surfaces and a terraced appearance. Some of these microcrystals also exhibit pits of hundreds of nanometers at their lateral surfaces, as shown in the inset in Figure 3d. In this range of high current values (5.8–6.0 A), which correspond to temperatures around 1200–1250 °C, only minor changes are observed in the samples apart from an improved homogeneity of the microcrystals formed during the JH treatment as the current increases. In general, the microcrystals' morphology resembles those obtained by the VS method at equivalent synthesis temperatures (Figure 2), although with smaller dimensions. Besides, the presence of pits and a terraced appearance is observed for both VS and JH methods.

To study the influence of the duration of the treatment, samples grown via JH with variable duration from 5 s to 10 min and a constant current of 5.5 A (∼1150 °C) were analyzed by SEM. In Figure 4a, corresponding to growth times of only 5 s, large flat grains can be observed together with some other smaller grains, some of which start to exhibit a faceted appearance, similar to those appreciated in the first stages of microcrystal formation (Figure 3b). By increasing the duration of the JH treatment up to 3 min, microcrystals with dimensions of around 5 μm and well-faceted surfaces are observed in a higher concentration, as shown in Figure 4b, similar to those obtained by the VS method (Figure 2). The increased duration of the JH process from some seconds to few minutes promotes not only the growth of microcrystals but also the formation of some nanostructured features at their lateral surfaces. Longer treatments of 5 min led to similar faceted microcrystals with dimensions of around 5 μm, eventually exhibiting pits at their surfaces (see the inset in Figure 4c) as well as a terraced appearance. No significant variations are induced by longer treatments of 10 min apart from an improved homogeneity in the morphology and dimensions of the microcrystals (Figure 4d).

**Figure 4:**
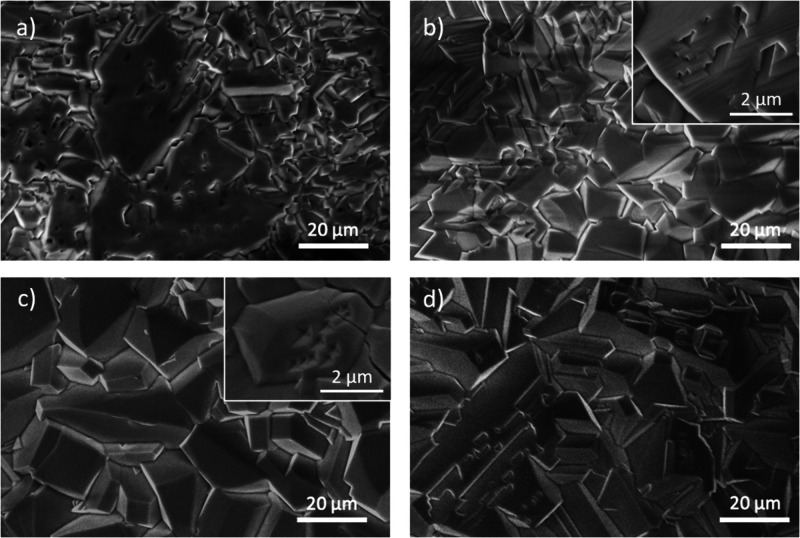
SEM images of (a) JH-1150-5s, (b) JH-1150-3m, (c) JH-1150-5m, and (d) JH-1150-10m from treatments at a constant current of 5.5 A and increasing growth duration.

Our observations indicate that current values higher than 5 A, which correspond to current densities and temperatures higher than ∼10^4^ A/cm^2^ and 1000 °C, respectively, and treatment durations of around 3 min are optimal parameters to synthesize microcrystals with relatively homogeneous dimensions and morphology. Importantly, the JH method results in samples with similar features as compared to samples grown by the conventional VS process that contrarily involves a much longer duration (hours) and, consequently, a significantly higher energy consumption. Another important advantage is that the rapid synthesis would also accelerate the material optimization.

Despite the morphological similarities, the faster oxidation and growth processes involved in the JH method could imply differences in the defect structure of the as-grown NiO compared to the VS samples. A set of XRD, EDS, and Raman spectroscopy techniques have been employed to evaluate the composition and crystalline quality of the NiO samples as well as to gain insights into the physical phenomena involved in the JH process.

After the JH process, the oxidized layer formed around the metallic Ni wire was detached for XRD analysis. Figure 5a shows XRD patterns acquired from samples JH-1200-3m and VS-1200 as representative of the JH and VS methods, respectively. In both cases, the diffractograms show peaks that correspond to crystalline NiO with a rock-salt structure (space group *Fm*3̅*m*, JCPDS Nno. 47-1049) dominated by the (200) reflections at 43.3° in both VS and JH samples. Taeño et al.^19^ observed that microcavities in NiO grown by VS show lateral faces that correspond to (100) and (111) family planes. Here we can observe that the (111) reflections at 37° are more pronounced in the samples grown by JH at a similar temperature (1200 °C). In addition to the characteristic NiO diffraction peaks, other contributions related to metallic Ni are observed in the sample fabricated by JH (marked by green dots in Figure 5a), which are likely due to unoxidized Ni traces from the wire incorporated during the sample preparation for XRD measurements.

**Figure 5:**
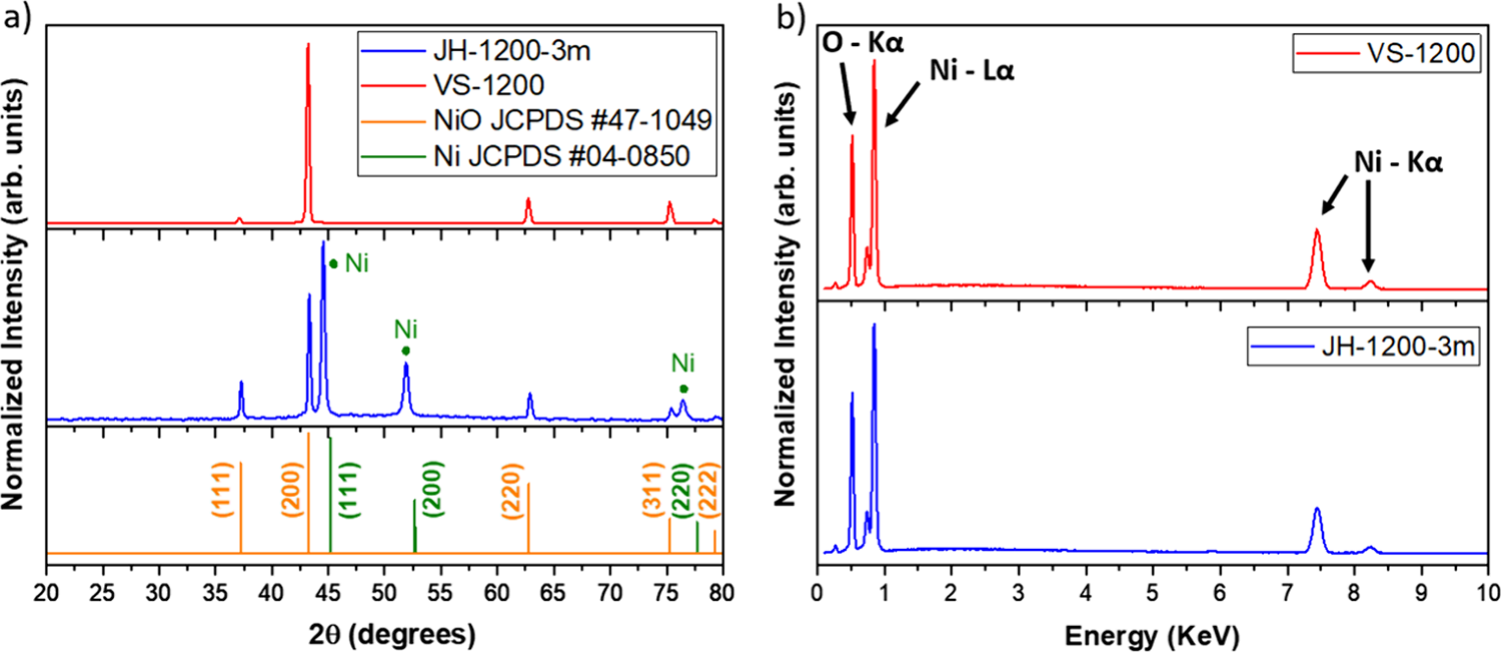
(a) XRD patterns and (b) EDS spectra from samples VS-1200 and JH-1200-3m.

The compositional EDS analysis performed at 20 keV shows only X-ray signals from O and the Ni elements from both samples fabricated by JH and VS, with a negligible presence of other impurities within the sensitivity limits of the technique. A representative EDS spectrum acquired from the sample JH-1200-3m is shown in Figure 5b, including the VS counterpart (VS-1200) for comparison. The average amount of Ni and O in the samples is consistent with the expected nominal values for NiO; however, the Ni/O ratio is less in the JH samples (∼1.1) as compared to the VS ones (∼1.2–1.3).

To get a deeper knowledge of the NiO formation during the JH process, a thorough analysis of the cross section has been performed. To proceed, small pieces of the NiO crust formed in the middle region of the treated Ni wire were carefully detached for inspection.

Figure 6a shows a cross-sectional SEM image of the NiO crust from one representative sample, JH-1150-3m, synthesized by the JH method at a temperature of around 1150 °C for 3 min. Two well-differentiated regions can be identified. The bottom side of Figure 6a shows the inner region, which is in close contact with the Ni wire during the JH process. On the upper side is the outer region, which is exposed to atmospheric air during the heating. The growth zone in close contact with the Ni wire (inner region) exhibits a thickness of around 3 μm, while the thickness of the outer region can vary between 7 and 9 μm. Furthermore, the inner region is characterized by small and faceted crystals with sizes in the range of hundreds of nanometers to a few microns, as well as the presence of small pores (inset in Figure 6a), while the outer region is formed by larger microcrystals, with dimensions of around 5 μm, and a more compact and columnar appearance. A similar duplex layer growth formed by large columnar grains in the outer region and equiaxed finer grains in the inner region in close contact with the metal has been reported for NiO and other metal oxide systems annealed at high temperatures by conventional thermal oxidation.^23,24^ It should be noted that different growth conditions are associated with these two regions. On the one hand, poorer oxygen exposure and higher temperatures are expected in the inner region. On the other hand, the outer region is directly exposed to air at room temperature, so it is reasonable to expect an abrupt temperature gradient across the wire during the JH process, as well as variable oxidation phenomena.

**Figure 6:**
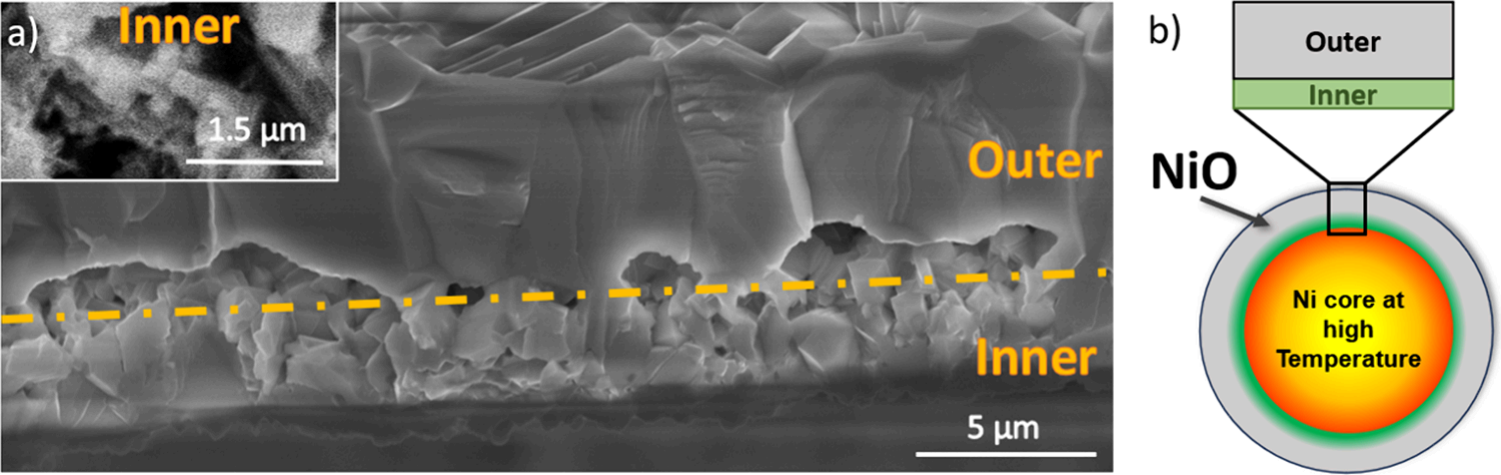
(a) Cross-sectional SEM image of sample JH-1150-3m and (b) schematic picture of the Ni wire during Joule heating treatment differentiating the cross-sectional inner and outer regions.

### 3.2 Micro-Raman Analysis

In addition to the composition and crystalline structure of the materials, micro-Raman spectroscopy allows us to study the samples with micrometric resolution. It is important to mention that the study of NiO by Raman spectroscopy is not straightforward because Raman scattering is caused by lattice vibrations and deviations from the perfect rock-salt structure. Therefore, other inherent processes play an important role in the phonon structure due to the intriguing NiO nature. Some of the reported processes involved in the NiO Raman signal correspond to surface optical phonons (SO), crystal lattice distortions induced by magnetic order, spin–phonon or magnon–phonon (1P + 1M) coupling, and magnon–magnon (M–M) coupling, among others.^25–30^ In this work, a comparative and comprehensive study between samples grown by the VS and JH methods has been performed. The position, intensity, and shape of the characteristic vibrational modes strongly depend on the synthesis process that determines not only the crystal dimensions but also the stoichiometry and structure of defects. Hence, this study can get insights into the understanding of the NiO Raman signal and its dependence on the synthesis parameters as well as achieve a deeper knowledge of the physical mechanisms involved in the growth of NiO microcrystals by the JH method. The Raman spectra of the untreated wire can be seen in Figure S2.

Figure 7 shows Raman spectra acquired with a 633 nm laser from different samples synthesized by the VS and JH methods. The spectra have been normalized with respect to the first-order modes at wavenumbers <700 cm^–1^. Figure 7a shows Raman spectra from samples grown by VS and JH (outer regions) at similar temperatures during the synthesis process. Two regions can be identified in the Raman spectra for all the analyzed NiO samples: first-order Raman modes from 300 to 660 cm^–1^ and second-order Raman modes from 660 to 1800 cm^–1^. In the low-wavenumber region, a first band associated with transverse optical (TO) modes, ranging from 320 to 464 cm^–1^, can be observed together with the longitudinal optical (LO) mode located around 544 cm^–1^. Overtones or second-order modes are observed at wavenumbers greater than 660 cm^–1^. In this region, the 2TO mode at 713 cm^–1^, a combined LO + TO mode at 898 cm^–1^, and the 2LO mode at 1088 cm^–1^ are observed. Finally, the 2M mode, associated with the two-magnon scattering or magnon overtone, is peaking around 1482–1500 cm^–1^. The position and shape of the Raman modes observed in Figure 7a are in good agreement with those reported for NiO in the rock-salt structure by other authors,^28,29^ including those reported for NiO grown by VS,^19^ which confirm the good crystalline quality of the microcrystals synthesized by JH despite the fast growth process (only 3 min) at ambient conditions. Both VS and JH samples exhibit similar NiO vibrational modes; however, some significant variations are observed in their relative intensity. The LO mode at 544 cm^–1^, related to Ni^2+^–O stretching oscillations,^27,28^ is dominant for all the samples grown by JH. On the other hand, VS samples exhibit second-order modes with higher relative intensity and a more complex structure on the first-order Raman modes, even exhibiting TO splitting in the sample VS-1200, usually associated with antiferromagnetic ordering in the NiO lattice.^25^ Similarly, 2TO and TO + LO modes are more well-defined in the VS samples as compared to JH, where these modes are weaker and barely resolved. By comparing the samples VS-1100 and VS-1200, we can notice that the Raman signal from VS samples is more sensitive to changes in the annealing temperature, contrary to the samples grown by JH, where their characteristic Raman spectra show minor changes for the same range of temperatures. Different authors have associated the observation of the first-order Raman modes with relaxation of the Raman selection rules due to NiO nonstoichiometry and additional structural defects in the samples.^31,32^ In addition, a reduction of the relative intensity of the 2M mode followed by a shift toward higher wavenumbers can be also attributed to a Néel temperature change due to biaxial strain via antiferromagnetic Ni–O super exchange interaction.^33^ Therefore, even though good crystallinity and similar morphological features are observed for the JH samples, these results indicate that the defect structure is quite different due to the unconventional fast oxidation mechanism in comparison to thermally grown NiO.

**Figure 7:**
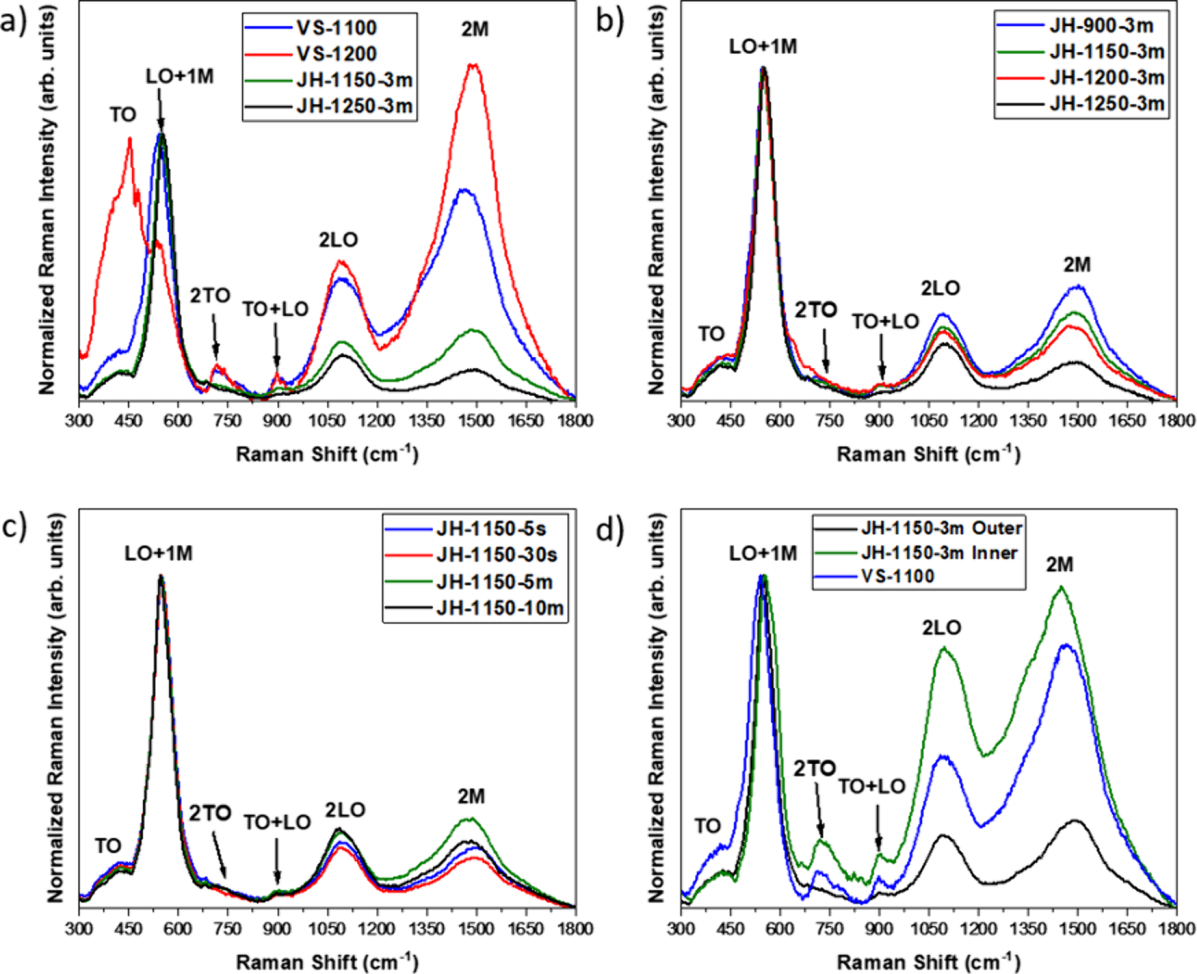
Raman spectra corresponding to (a) samples fabricated by VS and JH methods at similar temperatures and samples synthesized by JH at (b) variable currents and (c) variable durations. (d) Raman spectra acquired at the inner and outer regions of the crust of sample JH-1150-3m.

To analyze how the synthesis parameters affect the Raman characteristics, normalized spectra from samples fabricated by JH at different temperatures (varying the applied current) and constant duration (3 min) and samples fabricated by a constant temperature (or current) and varying the treatment duration are shown in Figure 7b,c, respectively. Contrary to VS samples, it is clear that the spectral features at the first-order Raman region (<700 cm^–1^) are more consistent for JH samples and that only minor variations can be appreciated at higher wavenumbers as a function of the growth parameters. By increasing the applied current and hence the synthesis temperature, a slight decrease in the relative intensity of the second-order modes (2TO, 2M) with respect to the first-order modes (TO, LO) is observed in Figure 7b. Besides, 2TO and TO + LO modes around 900–1000 cm^–1^ are generally less defined, to a greater extent for the sample grown at higher temperature (∼1250 °C). Surprisingly, even less noticeable are the variations as a function of the duration of the treatment (Figure 7c). In this case, only minor changes in the relative intensity of the second-order modes are observed, increasing their relative intensity with the treatment duration together with a slight shift (ca. 20 cm^–1^) of the 2M mode toward higher wavenumbers for treatments longer than 3 min. It is worth mentioning that well-defined Raman modes from NiO are observed even for the JH sample treated during 5 s, thus confirming the fast oxidation process.

Lastly, Figure 7d shows a comparison of the Raman spectra measured directly on the inner and outer regions of the NiO by flipping over a crust similar to the one shown in Figure 6. In this case, clear variations are appreciated in the Raman spectra from the inner and outer regions, likely due to the different growth conditions that result in a notably different structure of defects. Generally, the relative intensity of second-order modes (>700 cm^–1^) increases with respect to the LO mode for the inner region as compared to the outer side, where the LO mode dominates the Raman spectrum. In particular, the 2M mode dominates in the Raman spectrum from the inner region for sample JH-1150-3m, similarly to the VS-1100 sample, also included in Figure 7d for comparison. Therefore, the Raman spectra acquired from the inner region present, seemingly, similar features to those measured from the VS samples. Indeed, the 2TO and LO + TO modes are also more defined for both VS sample and the inner region of the JH sample. Qiu et al.^25^ reported a significant relative increase of the 2 M mode with respect to the LO mode as the oxygen content decreases in the annealed samples, possibly due to the related local Ni^2+^ symmetry conversion in the NiO lattice. In the present work, the inner region is not directly exposed to oxygen during the JH treatment as compared with the outer region, which is consistent with an oxygen-defective region. Additionally, diverse authors reported a change in color from black to green in NiO as the oxygen content decreases, in agreement with the color observed in the inner region (see Figure S3). It should be noted that a lower oxygen content involves variable oxygen and nickel vacancy concentrations, as well as Ni^3+^/Ni^2+^ ratio in the samples, which determine the physical properties and applications of NiO.^19,20^

These observations suggest that we explore in depth the characteristics of the inner/outer regions by combining spatially resolved spectroscopic techniques. Therefore, a detailed hyperspectral cross-sectional analysis was performed using both EDS and Raman spectroscopies.

Figure 8a shows a cross-sectional SEM image from sample JH-1150-3m as a representative sample fabricated by the JH method. The interface between the inner and outer regions is marked by a dotted line, showing similar duplex growth as described in Figure 6. So, in the following, the inner/outer regions correspond to the bottom/upper part of the cross-sectional images. EDS profiles of the yellow and green signals of the O and Ni signals, respectively, are included in Figure 8a. The compositional profiles acquired along the arrow marked in the image clearly indicate a decrease in the O/Ni ratio toward the inner region. Figure 8b displays EDS maps corresponding to the Ni–Kα (top) and O–Kα (bottom) lines acquired with a 20 keV e-beam. In this case, the probed inner region exhibits higher Ni, and correspondingly lower O signal, as compared to the outer region, in agreement with the EDS profiles. Similarly, an oxygen-poor innermost Cu_2_O layer in combination with an oxygen-rich outer CuO layer has been reported by Criado and Zúñiga in samples grown by similar resistive heating using a metallic Cu wire.^34^

**Figure 8:**
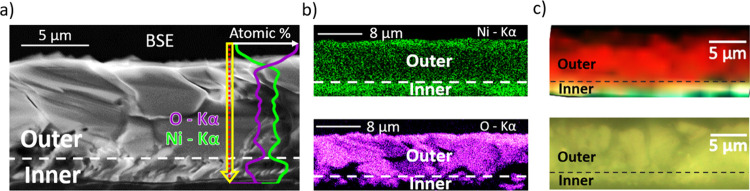
(a) Cross-sectional SEM image of the JH-1150-3m sample, including EDS profiles from O and Ni. (b) EDS mapping spectra corresponding to the Ni–Kα and O–Kα lines. (c) False color micro-Raman mapping (top) and its corresponding optical image (bottom) of the cross-sectional zone.

Due to the spatial resolution of the confocal microscope (∼1 μm spot size), hyperspectral Raman images were acquired in cross-sectional regions from the JH-1150-3m sample to complete the information obtained by local Raman spectra shown in Figure 7d. The top part of Figure 8c shows the false-color image corresponding to the optical image shown at the bottom. To generate the map, the signal from the LO + 1M (544 cm^–1^) mode was associated with red color, while the signals from 2LO (1088 cm^–1^) and 2M (1490 cm^–1^) modes were related to green and blue colors, respectively. Therefore, it is clearly observed that the second-order 2LO and 2M modes dominate in the inner region, while the first-order LO mode dominates the outer region, in agreement with the local Raman spectra shown in Figure 7d. As stated before, this increase of the 2M/2LO signal at the inner layer can be associated with a lower oxygen content in this region.^25^ The intersection of both regions is clearly identified and marked by a dashed line in Figure 8c that differentiates both domains. It can be appreciated that the width of the inner region remains nearly constant along the probed area. Thus, the Raman characteristics are reproducible and extended homogeneously for both inner and outer regions, and the thicknesses are proportionally similar in accordance with the observed SEM morphology and the color maps depicted in Figure 8b,c. These regions formed in the NiO overlayer clearly differ not only in the microstructure but also in the Ni/O ratio and physical properties owing to the variable oxidation conditions during the JH process. Some of the physical properties of NiO, such as the refractive index and the bandgap, strongly depend on the oxygen deficiency; therefore, the achievement of well-defined inner/outer regions with variable properties can be potentially exploited in devices such as optical resonators or energy filters, among others.

To address the mechanisms behind the growth of NiO via the JH process, diverse phenomena should be considered. Thermal oxidation mechanisms from metallic Ni commonly involve initial stages where chemisorbed oxygen dissociates at the metallic surface to form Ni–O^–^ groups as incipient NiO phases that lead to the growth of the oxide overlayer by the continuous contribution of electrons and Ni^2+^ ions from the underlying metal and oxygen form the surrounding atmosphere.^22^ Full oxidation of NiO is usually reached at 500–600 °C, while sintering and crystallization effects are promoted at higher temperatures, in agreement with our observations, although these processes can be influenced by other factors such as the presence of impurities and defects. Contrary to conventional thermal oxidation methods, during the JH process, both the high temperature and the continuous current flow can favor and accelerate oxygen dissociation phenomena at the metallic surface, leading to the rapid nucleation of defective NiO, at the initial stages of the process. This can be confirmed by the Raman signal from sample JH-1150-5s (Figure 7c) treated for only 5 s, where vibrational modes from NiO are clearly observed. Additionally, the thickness of the NiO layer achieved after a few minutes by the JH method is comparable to that formed at similar temperatures by conventional thermal oxidation over several hours, which also indicates a quite faster oxidation rate for the former. Regarding the formation of the duplex layer growth (Figure 6), the high nucleation rate at the initial stages of the oxidation process could promote the growth of small equiaxed grains, while the development of larger columnar grains from the initial grains becomes more favorable as the oxidation proceeds, specially under the beneficial oxidation conditions at the outer layer as compared to the more-defective inner one.

According to Wagner’s theory,^35^ nickel oxidation should follow a parabolic rate mainly controlled by Ni diffusion, as oxygen diffusion in NiO is much lower in comparison, in the range of temperatures employed in this work.^36,37^ Besides, at temperatures above 1000 °C, the oxidation kinetics should be governed by lattice diffusion, while grain boundary diffusion should dominate at lower temperatures for NiO.^36,37^ Therefore, during the formation of NiO from the metallic wire, Ni ions should migrate toward the outer layer via single or double Ni vacancies, while oxygen should be incorporated from gaseous species, possibly by stress-induced fissures and defects in NiO, leading to an oxygen-poor innermost layer, as that observed in Figure 8. However, Wagner’s theory of oxidation and thickening of the oxide layer does not consider the enhanced electromigration and variable ionic diffusion phenomena that occur during the JH process owing to the thermal stress gradient and the intense electric current flowing, which are suggested as the main mechanisms underlying the rapid oxide growth. In addition, the defect formation and migration activation energies involved in the oxide growth process can be influenced by the continuous current flow, which also induces local electric and magnetic fields in the wire during the formation and growth of NiO layers. These aspects can alter the kinetics of the oxidation growth, promoting as well complementary physical mechanisms in the scale of fast-growth processes. Moreover, in addition to thermal expansion, variable stress occurs at the oxide layer and the oxide/metal interface during the JH process. As the NiO growth occurs faster in the JH process, larger tensile stress should be induced in that case as compared to conventional thermal oxidation methods. In that sense, the possible detachment of the metal-oxide interface, where voids and pores can be found, should be also considered during the JH method, as this effect can alter the oxidation kinetics. Some works reported that the large compressive stress in NiO can lead to a decrease in the ionic diffusion coefficients that can finally reduce the oxide growth rate,^38^ while Ni migration from the metallic wire should be hindered as the NiO layer initiates to detach.

Stoichiometric NiO is commonly an insulating oxide with low electrical conductivity that can be increased by the generation of nickel vacancies and/or oxygen interstitials leading to a higher amount of Ni^3+^. Indeed, the optical and electrical properties have been extensively studied as a function of the oxidizing atmosphere or the doping process.^39^ However, good control of the defect structure, even by conventional methods, is still a challenging task. In this work, the EDS results point out that the O/Ni ratio at the inner and outermost layers formed during the JH process is different, and Raman spectroscopy confirms that the defect structure, as seen by the characteristic spectral features, should lead to different physical properties and therefore different applicability. The high electron flow and the rapid heating and cooling rate associated with JH could also be key points since the high current switches on the NiO growth at a desired temperature and the rapid cooling freezes the crystalline structure, so the remaining defects have to stabilize naturally through the most probable diffusion mechanisms. Our results suggest that improved control of the defect structure can be achieved by applying a high current flow during NiO growth.

As an additional advantage of the JH method, once the outer layer is removed, the initial metallic wire can be reused, thus improving the sustainability of the process. It is worth mentioning that despite the setup operating at ambient pressure, the JH process can be sophisticated by a controlled environment as well as by the presence of external electric or magnetic fields, which could give rise to unconventional crystal geometries due to field induced forces, adding versatility and room for improvement to the synthesis route. Furthermore, Rodríguez et al. reported that the application of an external electric field during the JH process can promote the diffusion and oxidation phenomena.^17^ Moreover, due to the high temperature reached by the wire, future work will be focused on the JH assisted growth of micro- and nanostructures of other relevant oxide-based binary and ternary semiconducting materials.

The achievement of a deeper understanding and control of the grain growth and microstructure has become a key issue in material science. Regarding conventional thermal annealing processes, including the VS method, mechanisms such as crystal nucleation, grain growth, atom diffusion through grain boundaries or via lattice, boundary migration, and crystal formation, among others, have been deeply analyzed and continuously revisited for metals and ceramics.^40^ Some of these processes can be governed by the presence of defects and Wulff construction. Deviations from the conventional grain growth have been commonly associated with variations on the driving forces that can involve atom diffusion or attachment as well as defect-assisted mechanisms. In this case, the JH process involves very fast oxidation and grain growth processes, in some cases analogous to those occurring during the VS method but governed by rapid nucleation and crystal growth mechanisms. These high-speed mechanisms can lead to variations in the kinetics of growth and the driving forces involved in microstructure evolution. Actually, the electromigration phenomena characteristic of the JH method, owing to the high current flow, can alter the diffusion mechanisms and hence the grain growth behavior. In some cases, this fast JH can lead to the formation of metastable phases, as recently reported by Rodríguez et al.,^41^ which paves the way to further improvements based on the JH method. This work can add novelty in the understanding and widening of the physical mechanisms involved in the grain growth and microstructure phenomena, thus providing alternative synthesis techniques that can enrich the materials science and technology fields.

## 4 Conclusions

To summarize, samples grown by both VS and JH methods at similar temperatures generally result in NiO microcrystals with similar morphological features. A reasonable parallelism has been shown facing samples grown from metallic Ni precursors by both synthesis methods. The main morphological difference observed is related to the microcrystal size, with smaller crystals in samples synthesized by JH. Similar results were also observed by XRD, with (200) and (111) being the dominant reflections for both JH and VS samples. In addition, the Raman signal from JH samples is dominated by LO modes without significant variations despite the changes in the growth conditions, contrary to the VS samples, where the Raman signal is more sensitive to changes in the growth parameters. Thus, we can suggest that the JH method is comparable to VS in terms of crystalline quality and morphology, with the great advantage of reducing the growth time by 2 orders of magnitude from more than 10 h down to only a few minutes, involving, at the same time, much lower energy consumption, economical saving, and scaling-up potential, which are some of the strengths of the JH method. Cross-sectional SEM, EDS, and Raman spectroscopy analysis confirms the presence of an oxygen-defective inner region in the NiO grown by JH, with dimensions around 3 μm and formed by small NiO crystals, while the outer region formed by larger grains exhibits a higher oxygen content. The relative increase in the 2M mode and second-order modes with respect to the LO one observed in the Raman signal of the inner region also confirms the related higher oxygen deficiency and the variation in the Ni^2+/^Ni^3+^ ratio as compared to the outer region. Hence, variable microstructure, composition, and physical properties can be promoted in the inner and outermost NiO layers grown by JH. The mechanisms underlying the JH process have been discussed and compared to those related to the VS method, which commonly involves conventional crystal nucleation, atomic diffusion, and grain growth. On the other hand, JH process involves very fast oxidation process, in some cases similar to those associated with the VS method but governed by rapid nucleation and crystal growth. Besides, the high current flow associated with the JH process also involves atomic diffusion and electromigration phenomena, exclusive of the JH method, owing to the intense electric current flowing, which can alter the kinetics of the grain growth. Additionally, since the metallic Ni wire during the JH treatments is simultaneously the precursor and heat source, the NiO growth environment could be locally manipulated by external fields applied during the JH process. Therefore, there is still room for improvement in tailoring both the morphology and physical properties of the grown crystals, thus adding versatility to this synthesis method. The results described in this work can be extended to other oxide micro- and nanomaterials with scaling-up potential as well as be used to optimize the JH process by further improvements.

## Supplementary Material



## Notes

Characterization of the untreated Ni wire (SEM and Raman) and optical images from the inner and outer region of the treated wire (PDF)
