# Supplementary material for: Micro- and Nanocrystalline NiO Synthesized by Joule Heating and Thermal Oxidation Methods: A Comparative Study
Source: Cryst Growth Des. 2025 Feb 6;25(4):1101–10. doi: 10.1021/acs.cgd.4c01439 (PMC12160586; doi:10.1021/acs.cgd.4c01439)
Supplement: Supplementary file 1 [file cg4c01439_si_001.pdf]

Supporting Information:

Micro- and nanocrystalline NiO synthesized by Joule heating and thermal oxidation methods: a comparative study

Diego J. Ramos-Ramos, G. Cristian Vásquez, and David Maestre*

Departamento de Física de Materiales, Facultad de CC. Físicas, Universidad Complutense de Madrid, 28040, Madrid, Spain

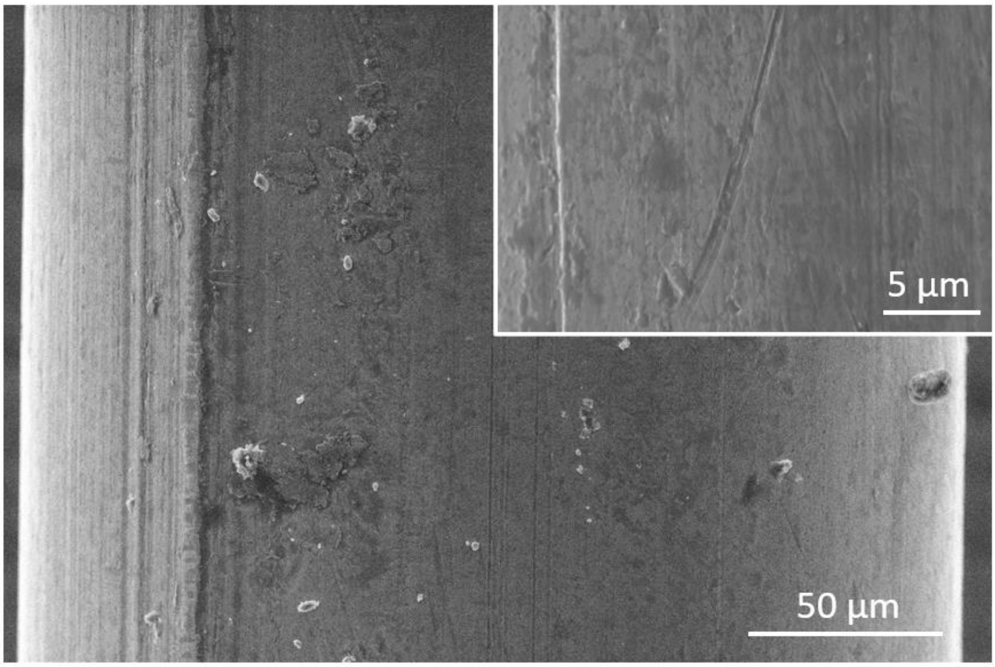

Figure S1. SEM image of the untreated Ni wire with a detail of the morphology of the surface (inset).

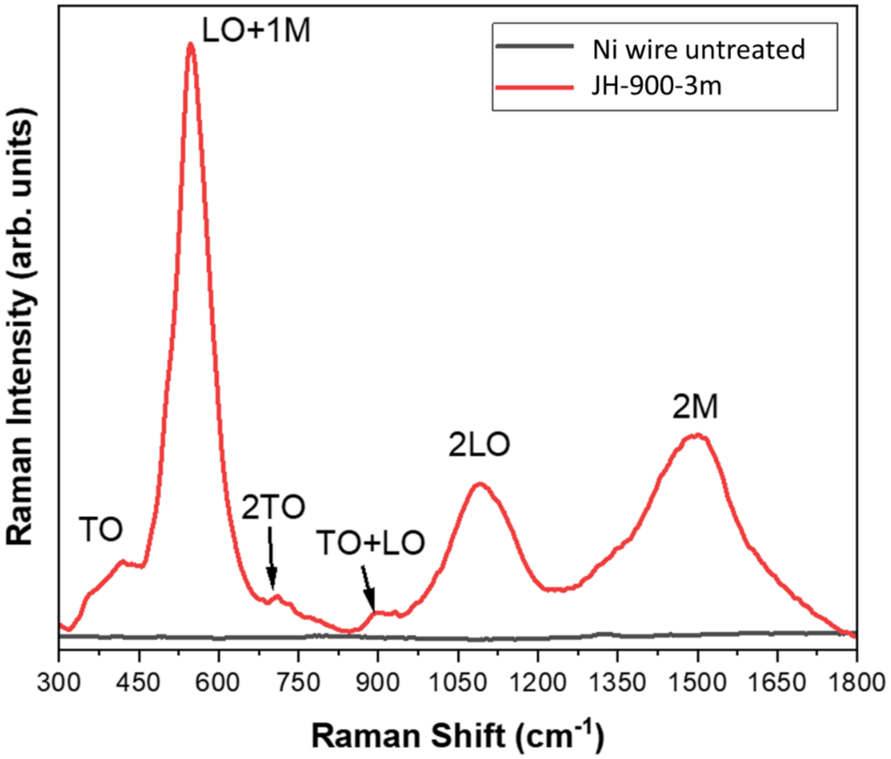

Figure S2. Raman spectra from the Ni wire before JH treatment and after treatment at 900 ºC during 3 min (JH-900-3m) using a 633 nm laser as excitation source.

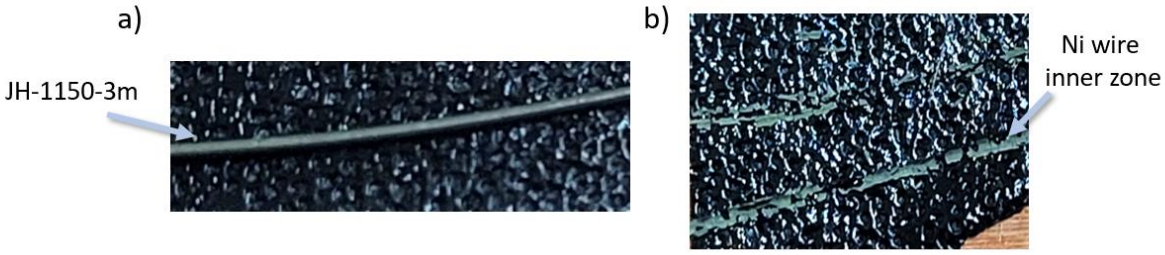

Figure S3. Optical images from treated wires a) JH-1150-3m before detached the surface and b) after being detached. It can be observed the difference in colour between the outer part (black) and the inner part (greenish).
